# Missed opportunities to increase efficiency of monoclonal antibody development using hybridoma technology and mice as the source animal

**DOI:** 10.3389/fimmu.2024.1443726

**Published:** 2024-08-12

**Authors:** Esha Chakravarty, Mehmet T. Dorak

**Affiliations:** School of Life Sciences, Pharmacy & Chemistry, Kingston University London, London, United Kingdom

**Keywords:** monoclonal antibody development, mice, BALB/c, hybridoma, histocompatibility, MHC restriction, antigen presentation, Th2 immune response

## Introduction

A tumor antigen must be sufficiently immunogenic to induce antibody production, and immunogenicity is determined by the strength of presentation by major histocompatibility complex (MHC)-encoded histocompatibility antigens. Not each peptide has sufficiently strong affinity to each MHC antigen, and therefore antigen presentation and subsequent immune response is dependent on the histocompatibility antigen present in the host animal. By increasing the likelihood of effective presentation and resulting immune response, heterozygosity for histocompatibility antigens enhances the chances for protection from infectious diseases and cancer as originally shown in animal models (1) and also in humans (2). Here, we want to highlight these facts for the benefit of scientists who want to improve the efficiency of their monoclonal antibody development programs using immunization of animals followed by hybridoma technology.

Immunization of laboratory animals, usually mice, with known or yet unknown immunogens, like cancer antigens, is the most common source of monoclonal antibodies to use for diagnostic or therapeutic purposes (3, 4). Once the animals produce monoclonal antibodies, the production at scale is most commonly achieved by the hybridoma technology (5). This is not a fail-proof method and a lot of modifications have been described, resulting in different immunization protocols that aim to stimulate the immune response in animals (6). The focus has been to increase the immunogenicity of antigens (7) and with success (8).

## Missed opportunities

The production of antibodies, however, could also be enhanced at the source by careful choice of the animals used. It has been a convention to use BALB/c mice because of its unique superiority in mounting immune response to immunological challenges (9), mainly due to being a Th2-type strain (10), which is particularly attractive if the aim is to induce humoral immune response. However strong the capacity of producing antibodies may be, a major determinant of antibody response is antigen presentation, which is governed by the MHC loci (H-2 in mice and HLA in humans) (11, 12). What remains unappreciated is that being an inbred mouse strain, BALB/c mice are homozygous at the entire H-2 haplotype (H-2^d^ haplotype), i.e, in each and every H-2 locus. This means that BALB/c mice, even though equipped with a strong humoral immune (antibody) response capacity, are restricted only to respond to antigens that can be presented by the H-2 loci of the H-2^d^ haplotype. A review of the development of all FDA approved therapeutic monoclonal antibodies has revealed that the majority are mouse antibodies developed using hybridoma technology which predominantly immunizes BALB/c mice to generate antibodies (13–16). A selection of such antibodies developed with hybridoma technology are presented in **Table 1**. The approach to immunize mice from different mouse strains to enhance the diversity of the resulting antibody repertoire has not been universally adopted although there are anecdotal examples in response to pathogens and vaccine development (9, 19, 20). This means we have only recorded success for maybe a small fraction of potential antibodies which could have been generated if multiple strains of mice with different H-2 haplotypes were used or even better, a set of H-2-congenic BALB/c mice were used, which are readily available (21). Another approach would be the use of obligatory heterozygote progeny of inbred mice (like the F1 generation of BALB/c and C57BL matings (21) or even progeny of H-2-congenic BALB/c mice to benefit from Th-2-dominant immune response in BALB/c, and the fact that many of the myeloma cells used for fusion to develop hybridomas have the same BALB/c origin (14).

**Table 1:** A selection of FDA / EMA-approved Monoclonal Antibodies Identified Using BALB/c Mice Immunisation and Hybridoma Technology^a^.

Brand name	Target	Antibody Type	Indication	Application	Year of Approval
Satumomab (OncoScint)	Tumour-associated glycoprotein (TAG-72)	Murine IgG 1	Colorectal or ovarian cancer	Diagnostic	1992
Capromab (ProstaScint)	Prostate Specific Membrane Antigen (PSMA)	Murine IgG1κ	Prostate adenocarcinoma	Diagnostic	1996
Nofetumomab (Verluma)	Carcinoma associated antigen	Fab fragment of murine IgG2b	Small cell lung cancer	Diagnostic	1996
Rituximab MabThera, Rituxan	CD20	Chimeric IgG1	Non-Hodgkin lymphoma	Therapeutic	1997
Trastuzumab (Herceptin)	HER2	Humanized IgG1	Breast cancer	Therapeutic	1998
Ibritumomab tiuxetan (Zevalin)	CD20	Murine IgG1	Treatment for relapsed or refractory, low grade or transformed B cell non-Hodgkin's lymphoma	Therapeutic	2002
Tositumomab and iodine 131 Tositumomab (Bexxar)	CD20	Murine IgG2a	Non-Hodgkin lymphoma	Therapeutic	2003
Bevacizumab (Avastin)	VEGF-A	Humanized IgG1	Colorectal cancer	Therapeutic	2004
Besilesomab (Scintimun)	NCA-95	Murine IgG1	Inflammatory lesions and metastases	Diagnostic	2010
Trastuzumab emtansine (Kadcyla)	HER2	Humanized Ig1 (Antibody-Drug Conjugate)	Breast cancer	Therapeutic	2012
*Mouse mAbs approved for indications other than cancer*						
Muromonab-CD3 (Orthoclone OKT3)	CD3	Murine IgG2a	Reduce rejection in patients with organ transplants	Therapeutic	1986
NeutroSpec (Fanolesomab)	CD15	Mouse labelled with radioisotope, technetium-99m (99mTc)	Appendicitis	Diagnostic	2004

a Adapted from Refs (4, 16–18).

The argument above may be extended to genetically engineered mouse models (GEMMs), which use knock-out/transgene method to replace the genes for immunoglobulins with human counterparts to produce humanized monoclonal antibodies upon immunization (22, 23). GEMMs are transgenic mice with humanized humoral immune systems (24). They have been used for production of immunomodulatory monoclonal antibodies as those used in cancer immunotherapy or autoimmune disease treatments (17), and anti-viral antibody production (25). Seven human mAbs developed using GEMMs are FDA approved (17). Even though, GEMMs are not frequently used for novel antigen discovery, most of these genetically humanized mouse models are derived from the C57BL/6 strain due to its well-characterized genome, robust immune response, and suitability for genetic engineering. However, some platforms may use BALB/c or a combination of strains to leverage specific characteristics beneficial for producing human antibodies. Both these strains are inbred mice and homozygous for H-2 haplotypes. This means the same arguments regarding the likelihood of presentation of novel antigens by H-2 also apply to GEMMs if these mice are used to develop monoclonal antibodies against novel cancer antigens.

## Discussion

It appears that there is a missed opportunity to increase the chances of obtaining many more monoclonal antibodies with potential to be used in diagnostic and therapeutic fields not only in cancer but also in vaccine development. By using congenic strains of BALB/c with different H-2 haplotypes (H-2^d^, H-2^b^, H-2^k^), an expanded antigen presentation capacity would naturally result in a large number of new monoclonal antibodies to be identified for clinical use. Most of the currently FDA-approved monoclonal antibodies have been produced by hybridoma technology (17), and mainly for cancer treatment (4) using H-2 haplotype homozygous mice. It is quite possible that many novel cancer antigens may have been missed due to lack of their presentation by the H-2 antigens of the inbred mice used. The bottom line is that techniques that used a single strain of inbred mice homozygous for an H-2 haplotype have a limited potential to discover novel antigens and many more antibodies could have been discovered to be tested for diagnostic and therapeutic potential.

The untapped potential highlighted in this paper may be exploited to identify many more cancer-specific monoclonal antibodies that may have been missed before by taking different approaches. These may be repeating the same experiments with immunologically more diverse mice or set of mice, or trying some of the more modern approaches that do not depend on hybridoma technology (17).
